# Elevated pulmonary arterial pressure and altered expression of *Ddah1* and *Arg1* in mice lacking cavin-1/PTRF

**DOI:** 10.1002/PHY2.8

**Published:** 2013-06-07

**Authors:** Karl Swärd, Mardjaneh K Sadegh, Michiko Mori, Jonas S Erjefält, Catarina Rippe

**Affiliations:** Department of Experimental Medical Science, Biomedical Centre, Lund UniversityBMC D12, SE-221 84, Lund, Sweden

**Keywords:** *Bmp4*, caveolin, *Foxc2*, *Lpin1*, nitric oxide, plau

## Abstract

Caveolae are invaginations in the plasma membrane that depend on caveolins and cavins for maturation. Here, we investigated the pulmonary phenotype in mice lacking cavin-1. Bright field and electron-microscopy showed that the cavin-1-deficient mice lacked caveolae in the lung, had an increased lung tissue density, and exhibited hypertrophic remodeling of pulmonary arteries. The right ventricle of the heart moreover had an increased mass and the right ventricular pressure was elevated. A microarray analysis revealed upregulation of *Arg1* and downregulation of *Ddah1*, molecules whose altered expression has previously been associated with pulmonary arterial hypertension. Taken together, this work demonstrates vascular remodeling and increased pulmonary blood pressure in cavin-1 deficient mice and associates this phenotype with altered expression of Arg1 and Ddah1.

## Introduction

Pulmonary arterial hypertension (PAH), a condition associated with high morbidity and mortality, is defined in man as an elevation of pulmonary arterial pressure at rest (>25 mm Hg) (Badesch et al. 2009). Similar to elevation of blood pressure in the systemic circulation (Heagerty et al. 1993), PAH results in remodeling of pulmonary arteries with increased media thickness (Mandegar et al. 2004). PAH can be a consequence of left heart failure and can be induced by hypoxia and certain drugs and toxins, but heritable forms also exist. Heritable forms are due to mutations in genes related to transforming growth factor β signaling, most often *BMPR2*, but occasionally also involving *ACVRL1*, *ENG*, and *SMAD9* (Machado et al. 2009). An important disease mechanism in acquired and idiopathic PAH is downregulation of the enzyme dimethylarginine dimethylaminohydrolase (Ddah, encoded by the genes *Ddah1* and *Ddah2* in mouse) which eliminates dimethylarginines, including asymmetric dimethylarginine (ADMA), from the circulation (Gorenflo et al. 2001; Arrigoni et al. 2003; Millatt et al. 2003; Kielstein et al. 2005). ADMA is an endogenous inhibitor of nitric oxide synthase (NOS) activity, and mice with one disrupted *Ddah1* allele have increased pulmonary arterial blood pressure (Leiper et al. 2007).

Recent work has uncovered mutations in caveolin-1, a protein involved in biogenesis of 50–100 nm large membrane invaginations referred to as caveolae, as an additional, albeit probably rare, cause of heritable PAH (Austin et al. 2012). This adds to work in mice that lack caveolin-1 and have elevated blood pressure in the pulmonary circulation (Maniatis et al. 2008; Wunderlich et al. 2008b; Zhao et al. 2002; Zhao et al. 2009). The mechanistic understanding of PAH in caveolin-1-deficient mice remains incomplete, but one proposed mechanism is tyrosine nitration of protein kinase G (PKG) (Zhao et al. 2009). This renders PKG inactive, presumably promoting pulmonary vasoconstriction, despite increased systemic nitric oxide (NO) levels. Long-term pharmacological or genetic NOS inhibition mitigates PAH in caveolin-1-deficient mice (Wunderlich et al. 2008a; Zhao et al. 2009), possibly because NO is a substrate in the PKG nitration reaction. A cell-permeable caveolin-1 peptide has been demonstrated to antagonize monocrotaline-induced PAH (Jasmin et al. 2006), suggesting pathogenic similarities between heritable and acquired forms of the disease.

Biogenesis of caveolae is a complex process involving proteins from several different families. Recent work has established a role of the protein PTRF/cavin-1 in formation of caveolae (Hill et al. 2008). Cavin-1-deficient mice have been generated and these were reported to have a metabolic phenotype with insulin resistance (Liu et al. 2008) as well as considerable perinatal lethality (Karbalaei et al. 2012). Here, we addressed the functional significance of cavin-1 in the lung by exploiting cavin-1-deficient mice. We demonstrate that these mice have altered lung structure, remodeled lung vessels, increased right ventricular weight, and elevated right ventricular pressure. In addition, a microarray analysis demonstrated altered levels of arginase 1 (Arg1) and Ddah1, enzymes involved in regulating NO production. Taken together, our findings show that ablation of cavin-1 leads to elevated pulmonary arterial pressure, and point to shared disease mechanisms between acquired and heritable forms of PAH.

## Materials and Methods

### Mice

Cavin-1-knockout mice were bred and genotyped as described (Karbalaei et al. 2012). All mice were housed in an animal care facility at Lund University on a 12:12 light:dark cycle and had access to food and water ad libitum. Newborn mice were sacrificed within 12 h of birth for the microarray experiment and the confirmatory RT-qPCR (real-time quantitative polymerase chain reaction), and adult mice (4–5 months) were used in the remainder of the experiments. No significant skew toward heterozygotes and wild types was seen at birth, contrasting with the situation at 4 weeks (Karbalaei et al. 2012). Comparisons were made between knockout (KO, ^−/−^) and wild type (WT, ^+/+^) littermate controls. All experiments were approved by the local (Malmö-Lund) ethics committee.

### Lung tissue preparation and histological analysis

Whole lung lobes (2–3 lobes per animal) were excised from adult and newborn mice and immersed in 4% paraformaldehyde. After dehydration, the tissues were embedded in paraffin and sectioned (4 μm). Masson trichrome-stained sections were analyzed to assess fibrosis. Mayer's hematoxylin and eosin-stained sections were analyzed for tissue density and pulmonary vessel media thickness. Stained sections were digitized with a slide-scanner (20×, ScanScope, Aperio Technologies, Inc., Vista, CA) and morphometric measurements were performed on the generated high-resolution images. The total cross-sectional area of each lobe was measured using the Aperio Positive Pixel Count Algorithm v.9 (Aperio Technologies) and the tissue density was expressed as a percentage of the total tissue area (excluding airspaces) per total lobe area (including airspaces). For each muscularized vessel, the outer perimeter of the media and the inner perimeter of the endothelium were outlined by manual cursor tracing and measured by the Aperio ImageScope software v.10 (Aperio Technologies). The diameters were calculated and used to determine the media thickness, which was defined as the distance (μm) between the outer and inner vessel diameters. Vessels were grouped according to their lumen diameters. Vascular lumen diameter was calculated as the sum of the maximum and minimum distances across the lumen divided by two. All histological analyses were performed in a blinded manner.

### Immunohistochemistry

Human distal lung tissue was obtained in association with lung transplantation for advanced chronic obstructive pulmonary disease (Lund University Hospital, Lund, Sweden). Informed consent and local ethical approval (Malmö-Lund ethical committee) were obtained. Paraffin sections were heated for 20 min at 60°C and antigen retrieved in EnVision™ FLEX Target Retrieval Solution (K8005, Dako, Glostrup, Denmark) in a Dako PT-Link module. Immunohistochemistry was performed with EnVision™ Peroxidase/DAB Detection System kit (K5007, Dako) using an automated immunostainer (DakoCytomation, Glostrup, Denmark). Endogenous peroxidase activity was quenched in 0.3% hydrogen peroxide for 10 min. Sections were incubated with a primary antibody directed against cavin-1 (dilution 1:300, ab48824, Abcam, Cambridge, MA) for 1 h and then with horse radish peroxidase (HRP)-linked secondary antibodies for 30 min. Immunoreactivity was visualized with 3'3-diaminobenzidine (DAB). After staining, sections were dehydrated in a series of ethanol, cleared in xylene, and mounted with Pertex (HistoLab, Gothenburg, Sweden).

### Cavin-1-reporter staining

The targeting vector used to generate cavin-1-knockout mice contained a lacZ/neo cassette (Liu et al. 2008). This allows for analysis of β-gal expression under control of the endogenous cavin-1 promoter. To examine β-gal expression, lungs were retrieved from heterozygous mice. Micro-dissection was performed in physiological buffer (HEPES-buffered Krebs solution) (Karbalaei et al. 2012), and lungs were fixed briefly (10 min) with 4% paraformaldehyde in phosphate-buffered saline (pH 7, room temperature). Staining was performed in a solution containing 150 mmol/L NaCl, 2 mmol/L MgCl_2_, 5 mmol/L potassium ferricyanide, 5 mmol/L potassium ferrocyanide, 40 mmol/L citric acid, 12 mmol/L sodium phosphate (pH 6.0), and 1 mg/mL X-gal (5-bromo-4-chloro-3-indolyl-beta-d-galactopyranoside, Sigma, St. Louis, MO) at 37°C. Tubes were inspected every 15 min for the appearance of indigo stain, and the reaction was stopped when sharp contrasts were apparent (15 min–24 h). Photographs were taken using an Olympus (SZ61) dissection microscope (nongraded 0.67–4.5× optical zoom) fitted with an Infinity 1 digital camera.

### Conventional and scanning electron microscopy

Lungs were rapidly excised and immersed 2.5% glutaraldehyde in 150 mmol/L cacodylate buffer (pH 7.4). After 24 h the tissues were transferred to cacodylate buffer and further postfixed in 1% osmium tetroxide for 2 h, block stained with uranyl acetate, dehydrated and embedded. Sections were cut and examined in a transmission electron microscope. Digital photos were analyzed using ImageJ (National Institutes of Health [NIH], Bethesda, MD). Samples for scanning electron microscopy were washed after fixation and then dehydrated, critical point dried and sputtered with palladium/gold. Samples were examined in a JEOL JSM-350 scanning electron microscope.

### Right ventricular pressure

Mice (6 WT and 6 KO) were anesthetized with 4% isoflurane (Isoflurane Forene; Abbot Scandinavia, Sweden) in room air in a small container and were transferred to a heating pad controlled by a rectal probe to keep temperature at 37±1°C (Temperature Control Unit HB 101/2; Panlab, Spain). After tracheotomy, anesthesia was controlled and maintained at 2% isoflurane by using a mouse ventilator (28025; Ugo Basile, Italy, tidal volume: 0.35 mL, frequency: 98/min). A thoracotomy was performed and a needle coupled to a pressure transducer was advanced into the right ventricle for pressure measurement using a polygraph (Model 7B; Grass Instruments, Quincy, MA).

### RNA isolation

Newborn cavin-1 KO and WT mice were euthanized by decapitation. Lungs were dissected and frozen in liquid nitrogen. Lung tissue from 10 newborn cavin-1 KO and WT mice was homogenized using a Qiagen Tissuelyser followed by RNA extraction with RNeasy Mini Kits (Qiagen, Valencia, CA) according to the manufacturer's protocol. The purity and concentration of the RNA was determined with an ND-1000 spectrophotometer (Nanodrop Technologies Inc. Wilmington, DE) and the integrity was determined using a bioanalyzer (Agilent 2100 Bioanalyzer, Santa Clara, CA).

### Microarray analysis

Mouse whole genome microarray, DirectHyb MouseWG-6 v2.0 (accession no. GLP6887, Illumina Inc., San Diego, CA) were from SCIBLU Genomics (Swegene Centre for Integrative biology, Lund University, Sweden) and used according to the manufacturer's instructions. The Basic Illumina chip and Experimental Quality Analyses were performed using the GenomeStudio software V2011.1.

### Real-time PCR

To confirm the microarray data we used the QuantiTect primer assays and QuantiFast SYBR Green RT-PCR kit (Qiagen) for the following genes: *Ddah1* (QT00149975), *Meox2* (QT00170268), *Shh* (QT00122479), *Wnt5a* (QT00164500), *Arg1*(QT00134288), *Gja4* (QT01062208), *Rab38* (QT00163660), *Traf1* (QT00142366), *Wisp1* (QT00111230), *Cdkn1a* (QT00137053), *Ddit4* (QT00250614), *Igfbp3* (QT00493332), *Nfkbia* (QT00134421), *Sema7a* (QT00173488), *Sesn1*(QT01053458), *Tnfrs11b* (QT00106757), *Bmp4* (QT00111174), *Dgkg* (QT001370398), *Gata5* (QT00132678), *Lims1* (QT00121590), *Meox1* (QT00107597), *Nrarp* (QT00262199), *Plau* (QT00103159), *Ppp1r1b* (QT00130970), *Rem1* (QT00100576), *Rhoe* (QT00128625), *Azgp1* (QT01057182), *Lpin1*(QT00139552), *Calm2* (QT01164772), *Angptl4* (QT00139748), *Fabp5* (QT01743770), *Nuak1*(QT01059303), *Ppp1r3b* (QT00285614), *Foxc2* (QT00252175). The quantitative PCR was performed on an Icycler IQ sequence detection system (Stratagene, M3000P, La Jolla, CA) with the following amplification cycles: 50°C for 10 min (reverse transcription) followed by denaturation at 95°C for 5 min before 40 cycles of: 95°C (10 sec), 60°C (30 sec). Samples were run as duplicates in a volume of 20 μL. Expression levels of the genes were calculated according to the Pfaffl method and glyceraldehyde 3-phosphate dehydrogenase, Gapdh (QT01658692), which was unchanged in the microarray experiment (fold change = 1.04, *P* = 0.8), was used as a reference gene.

### Western blotting

Tissues were frozen in liquid N_2_, pulverized using the Qiagen Tissuelyser (pre cooled to −80°C), and dissolved in sodium dodecyl sulfate (SDS) sample buffer (62.5 mmol/L Tris-HCl pH 6.8, 2% SDS [w/v], 10% [v/v] glycerol, 5% [v/v] mercaptoethanol) containing phosphatase and protease inhibitors (Sigma, St. Louis, MO). Protein concentration was determined using the Bio-Rad DC™protein assay. Using the Turboblot system (Bio-Rad, Hercules, CA), 20 μg protein was separated on a gel (4–15% or any KD, TGX Criterion, Bio-Rad) and transferred to nitrocellulose membrane (0.2–0.45 μm). Membranes were blocked in casein blocking buffer, washed in TBS-T (20 mmol/L Tris-HCl, 0.5 mol/L NaCl, 0.05% [v/v] Tween 20, pH 7.5), and incubated with primary antibodies (Ddah1 [ab82908, Abcam], Arg1 [GTX109242, Genetex, Irvine, CA], cavin-1 [ab48824, Abcam], cavin-2 [ab113876, Abcam], cavin-3 [16250-1-AP ProteinTech Group, Chicago, IL], caveolin-1, caveolin-2, caveolin-3 [610407, 610684, and 610421, BD Transduction laboratories, San Jose, CA]). Secondary antibodies (HRP-conjugated) and West Femto chemiluminescence substrate (Pierce, Rockford, IL) were used for detection in an Odessey Fc instrument (LI-COR Biosciences, Lincon, NE) (Sadegh et al. 2011). All bands were normalized to GAPDH (MAB374, Millipore, Temecula, CA) or HSP90 (610418, BD Transduction laboratories). These reference genes were not affected by ablation of cavin-1 as shown in the microarray experiment and as confirmed by western blotting.

### Statistics

Data are presented as means ± SEM. *P*-values were calculated using Student's *t*-test. *P* < 0.05 was considered statistically significant (*P < 0.05, **P < 0.01, ***P< 0.001). For the Illumina microarray experiment, probe summarization and normalization was performed using Robust Multi Array (RMA) analysis (Irizarry et al. 2003). Replicate genes were merged using median merge method. A SAM (significance analysis of microarrays) analysis was performed using TMEV v4.0 software to identify significantly differentially expressed genes between groups (Tusher et al. 2001). To compare the data obtained from the microarray with that from the RT-PCR a linear correlation analysis was performed using Graph Pad Prism.

## Results

### Cavin-1 expression in lung

**Figure 1:**
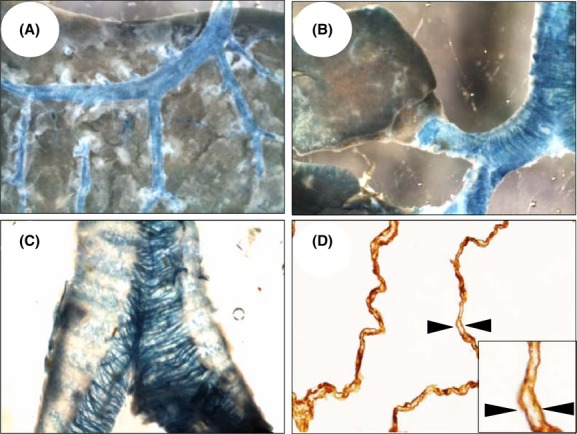
Cavin-1-expression in the lungs in mouse and man. (A–C) Cavin-1 reporter staining (blue/indigo) of lung structures; (A) shows that the pulmonary arteries are more strongly positive for cavin-1 than the surrounding parenchyma; (B) shows the trachea and main bronchi partially freed from lung parenchyma; and (C) shows the posterior wall of the trachea after cutting it open. Intense staining of the smooth muscle layer is apparent. Immunohistochemical staining of human lung (D) shows cavin-1 expression in alveolar septa. Inset in (D) highlights cavin-1 staining (brown) at the membrane (arrowheads).

Cavin-1 reporter staining was performed using heterozygous mice (Karbalaei et al. 2012). We found that the whole lung, including the parenchyma, was heavily stained after 2 h of substrate exposure (not shown). Shorter exposure (20 min) showed highest expression in the pulmonary arteries (Fig. 1A) as well as in airway smooth muscle (Fig. 1B and C). Immunohistochemistry of human lung tissue showed uninterrupted staining of alveolar septa with apparent membrane localization (brown, Fig. 1D). Cavin-1 was expressed in human lung endothelial and smooth muscle cells (data not shown).

### Cavin-1-deficient mice have reduced levels of caveolae-associated proteins and lack caveolae in the lung

**Figure 2:**
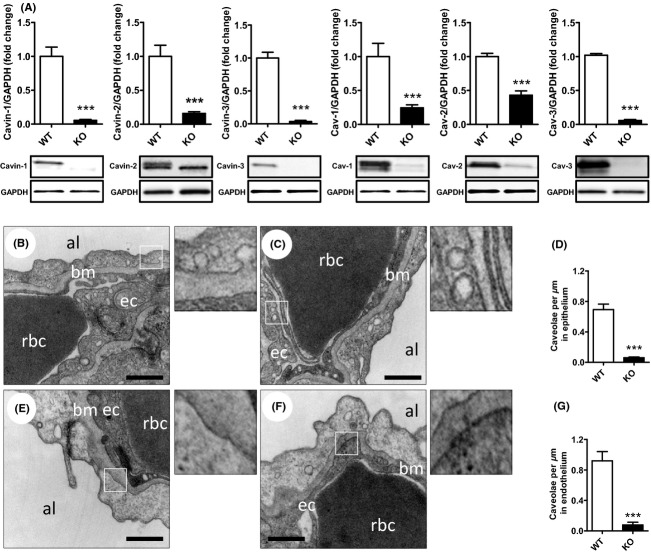
Expression of caveolae-associated proteins and caveolae abundance in cavin-1-deficient lungs. (A) Expression of cavins and caveolins in cavin-1^+/+^ (WT) and cavin-1^−/−^ (KO) mice. Electron micrographs of lungs from WT (B, C) and KO (E, F) mice. Areas within the white rectangles highlight membrane areas in epithelial cells (B, E) and endothelial cells (C, F) and are shown at a higher magnification to the right. Al, alveolar lumen; bm, basal membrane; ec, endothelial cell; rbc, red blood cell. Summarized data on the number of caveolae per μm membrane in alveolar type I cells and endothelial cells are shown in (D, G). Caveolae were counted on 107 electron micrographs (60K) from three WT and three KO mice. The total membrane length examined was 222 and 200 μm, for WT and KO, respectively.

Using lung lysates from adult mice, we examined the levels of caveolae-proteins by western blotting (Fig. 2A). Cavin-1 was absent as expected (*N* = 8–9) and cavin-3 expression was reduced (*N* = 3). The cavin-2 antibody detected two close bands, the higher of which was reduced (*N* = 9). Caveolin-1 was reduced (*N* = 6) as was caveolin-2 (*N* = 8–9) and caveolin-3 (*N* = 4). Transmission electron microscopy (Fig. 2B, C, E and F) confirmed lack of caveolae in alveolar type 1 cells and in lung endothelial cells (summarized data in Fig. 2D, G, *N* = 3).

### Lung structure is altered by cavin-1 deficiency in adult mice

**Figure 3:**
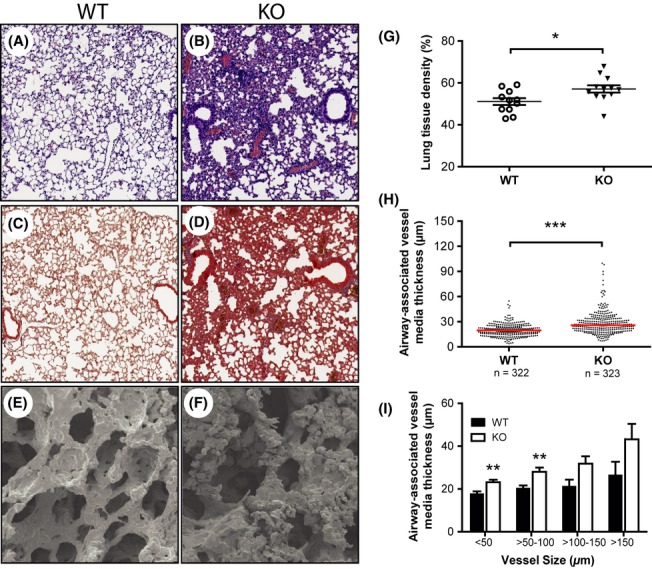
Structural changes in lungs from cavin-1 deficient mice. Hematoxylin–eosin (A, B) and Masson trichrome staining (C, D) of lung sections from wild type (WT) (A, C) and cavin-1^−/−^ (KO) (B, D) mice. The staining shows a greater tissue density in KO mice (B, D, summarized data in G) compared to WT (A, C, G). Scanning electron microscopy (E, WT; F, KO) shows an apparent increase in granularity in KO cells. (H) Media thickness in all lung vessels analyzed. All airway-associated vessels in (H) were grouped according to vessel size and means per diameter group and animal were calculated. This analysis (I) shows an increased media thickness in airway-associated blood vessels in cavin-1-deficient mice (<100 μm).

We next compared lung structure of adult mice using conventional histology. Focal changes in cellularity (compare Fig. 3A and B), resulting in a modest overall increase in lung tissue density (Fig. 3G, *N* = 11–12), were seen in KO mice. Masson trichrome staining did not support classical fibrosis (Fig. 3C and D). Changes were evident by scanning electron microscopy (Fig. 3E and F, *N* = 3) which also disclosed more granular cell appearances. Further analysis revealed that airway-associated vessels had an increased media thickness in KO mice (Fig. 3H, *n* = 322–323 measured vessels, *N* = 11–12 mice). Grouping of vessels according to diameter showed a significantly increased media thickness in blood vessels smaller than 100 μm (Fig. 3I). In newborn mice, we did not find any structural differences between WT and KO lungs (not shown).

### Right ventricular pressure is elevated in cavin-1^−/−^ mice

**Figure 4:**
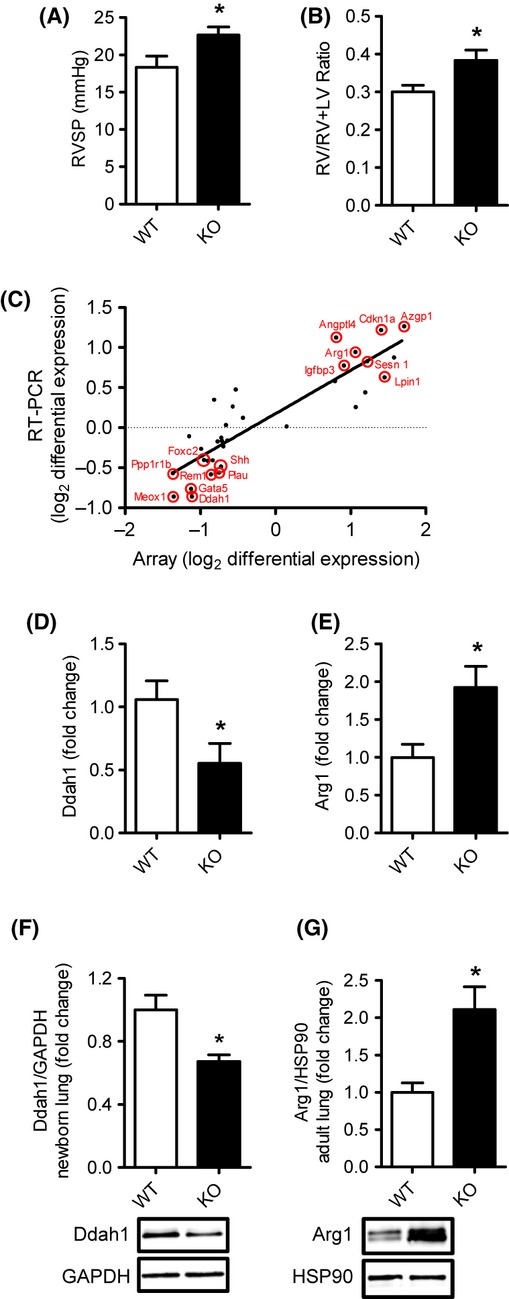
Elevated right ventricular pressure, increased right ventricular weight and altered expression of *Ddah1* and *Arg1* in cavin^−/−^ mice. Systolic right ventricular pressure an right ventricular weight is shown in (A) and (B), respectively. Measurements were made in adult mice. (C) A correlation between gene expression determined in a microarray experiments, done using newborn lungs, and confirmative RT-qPCR for 34 selected genes; 15 genes, indicated by red circles, were confirmed to be differentially expressed in cavin-1^−/−^ lungs by RT-qPCR. Confirmation of *Ddah1* and *Arg1* by RT-qPCR is shown separately in (D) and (E). Summarized data from Western blots for Ddah1 and Arg1 proteins are shown in (F) and (G). Lung lysates were from newborn mice in (F) and from adult mice in (G).

Because hypertrophic arterial remodeling is a hallmark of PAH, we measured the right ventricular systolic pressure (RVSP) in adult mice as an estimate of pulmonary arterial pressure. Compared to littermate controls an increased RVSP was seen in cavin-1-deficient mice (Fig. 4A, *N* = 6). We next dissected the heart and weighed the right and left ventricles. The ratio of right ventricular weight to total weight of the right plus left ventricles was elevated (Fig. 4B, *N* = 19–20).

### Differential expression of genes involved in nitric oxide production

To gain further insight into the pulmonary vascular changes, we ran a microarray experiment with lungs from six pairs of mice (GEO accession number GSE43561). We used newborn animals because we wanted to identify genes that are a cause of PAH rather than those that are a consequence of PAH. Forty-two genes were differentially expressed (*q* = 0) in KO compared to WT lungs. Except for cavin-1, none of the caveolins or cavins were different (*q* = 0). Fifteen genes (of 34 tested) were confirmed by RT-qPCR using 10 WT/KO lung pairs. There was a good correlation (*P* < 0.0001) between the microarray results and the RT-qPCR results (Fig. 4C, confirmed genes in red). The differentially expressed genes not only included those involved in lipid metabolism but also *Ddah1* (Fig. 4D) and *Arg1* (Fig. 4E).

Downregulation of *Ddah1* was confirmed at the protein level using lysates of newborn lungs (Fig 4F). The Arg1 protein level was not changed at birth (not shown), but when we used adult lung lysates an increased level was found (Fig. 4G). The Ddah1 protein level remained reduced in adult KO mice and was also found to be reduced in adult caveolin-1-deficient lungs (not shown).

## Discussion

This work establishes an impact of cavin-1 on pressure regulation in the pulmonary circulation. Right ventricular systolic pressure was robustly increased compared to WT littermate controls and accompanied by an increased right ventricular mass and remodeling of airway-associated blood vessels. Because PAH is a progressive disease and we used younger animals, one may predict a more full-blown PAH phenotype with aging. Since expression of caveolin-1 was partly maintained, cavin-1-knockout mice may constitute a more adequate model of heritable PAH due to mutations in caveolin-1 than do caveolin-1-knockout mice. This is because the disease-causing mutations cause a partial reduction of caveolin-1 (Austin et al. 2012), contrasting with the situation in caveolin-1-knockout mice where the protein is completely lacking.

In agreement with our current findings, thicker alveolar septa, hypercellularity, and elevated pulmonary pressure have been reported in caveolin-1-deficient mice (Drab et al. 2001; Razani et al. 2001; Zhao et al. 2002; Maniatis et al. 2008). The vascular remodeling of pulmonary vessels documented here is also consistent with increased pulmonary vascular resistance and reduced filling of small arteries in caveolin-1 knockout animals (Maniatis et al. 2008) and with PAH-induced vascular remodeling in humans with mutations in the *CAV1* gene (Austin et al. 2012). The lung phenotype in caveolin-1-deficient mice has occasionally been associated with fibrotic changes (Drab et al. 2001; Maniatis et al. 2008), and several studies have indicated an influence of caveolae and caveolin-1 on transforming growth factor β (TGF-β) superfamily signaling (reviewed by Meyer et al. 2013). Specifically, BMPR2 function was found to depend on the caveolin-1 β isoform (Nohe et al. 2005). This is of major interest given the role of this receptor in heritable forms of PAH. We did not detect fibrosis in cavin-1-deficient mice, and our array study did not support altered expression of *Bmpr2*, *Acvrl1* (ALK1), *Smad9*, or *Eng*. *Bmp4* was the only mediator in the TGF-β superfamily that was differentially expressed (down, *P* = 0.001, *q* = 0). This cytokine induces apoptosis in pulmonary artery smooth muscle cells through BMP receptor 2 (Yu et al. 2008), and further work is warranted to verify and understand the role of cavin-1 in this regard.

Two other genes, namely *Arg1* and *Ddah1*, were identified in our array experiment. Altered expression of these genes was confirmed at both mRNA and protein levels. Arg1 is an enzyme that converts l-arginine to ornithine and that has previously been found to increase in PAH (Hsu et al. 2007). l-arginine is a substrate for nitric oxide synthase. When l-arginine levels are limiting, eNOS uncouples to produce superoxide instead of NO (Pernow and Jung 2013). Upregulation of Arg1 may thus reduce l-arginine levels and lead to oxidative stress, which in turn may trigger smooth muscle cell proliferation. Because reactive oxygen and nitrogen species can further stimulate *Arg1* expression, a feed-forward circle of oxidative damage/proliferation may be envisioned. Arg1 may also contribute to pulmonary hypertension by stimulating NO-independent pathways such as the polyamine synthesis pathway. Recent work in caveolin-1 KO mice have reported increased lung expression of *Arg1* also in this strain (Aravamudan et al. 2012). Upregulation of arginase thus appears to be a conserved response to ablation of caveolae that may contribute to development of PAH.

*Ddah1* encodes an enzyme responsible for catabolism of asymmetric dimethyl arginine (ADMA). ADMA is a negative regulator of NOS and with increasing ADMA concentrations NO production decreases. The reciprocal changes in *Arg1* and *Ddah1* are thus expected to limit the increase of NOS activity widely reported for caveolae-deficient animals and cells (Razani et al. 2001; Zhao et al. 2002; Davalos et al. 2010) and reviewed by (Rahman and Sward 2009). This may occur in a temporal and spatial manner such that normalization of systemic blood pressure (Rosengren et al. 2006; Albinsson et al. 2007) occurs at the expense of an elevated pressure in the pulmonary circulation (this study; Murata et al. 2007; Maniatis et al. 2008; Zhao et al. 2002). In keeping with this hypothesis, several studies have demonstrated an inverse association between Ddah1 expression and acquired forms of PAH (Millatt et al. 2003; Kielstein et al. 2005). Indeed, disruption of only one allele of *Ddah1* reduced Ddah1 protein expression and activity by 40–45% and this was sufficient to cause PAH (Leiper et al. 2007). Small pulmonary arteries moreover responded more strongly to Ddah1 inhibition than did systemic arteries (9).

In conclusion, we show that mice lacking cavin-1 have elevated pulmonary arterial blood pressure, remodeled lung arteries, and an increased right ventricular mass. Our array experiment supports differential expression of at least a handful of genes centrally involved in lipid metabolism that warrant further study. We also demonstrate reciprocal changes in *Arg1* and *Ddah1* expression in the lung. These expression changes are identical to changes in acquired forms of PAH, suggesting a common disease mechanism. Pharmacological and vector-based strategies to treat PAH by targeting Arg1/Ddah1 may thus also find a place in therapy of heritable forms of the disease.
